# Studies on the Transmission of a Tigecycline Resistance-Mediating *tet*(A) Gene Variant from Enterobacter hormaechei via a Two-Step Recombination Process

**DOI:** 10.1128/spectrum.00496-22

**Published:** 2022-05-17

**Authors:** Runhao Yu, Zheng Chen, Danyang Li, Stefan Schwarz, Xinwei Wang, Xiang-Dang Du

**Affiliations:** a College of Veterinary Medicine, Henan Agricultural University, Zhengzhou, People’s Republic of China; b Institute of Microbiology and Epizootics, Centre for Infection Medicine, Department of Veterinary Medicine, Freie Universität Berlin, Berlin, Germany; c Veterinary Centre for Resistance Research (TZR), Freie Universität Berlin, Berlin, Germany; University at Albany, State University of New York

**Keywords:** tigecycline, resistance, *Enterobacter hormaechei*, *tet*(A) variant, transmission, antibiotic resistance, antimicrobial agents, dissemination, plasmid-mediated resistance, resistance genes

## Abstract

To investigate the contribution of a *tet*(A) variant to tigecycline resistance in Enterobacter hormaechei and the recombination events that occurred during transmission of this variant. MICs were determined by broth microdilution. *E. hormaechei* G17 was characterized by PCR, transfer assay, S1-PFGE, Southern blot hybridization, and WGS analysis. A *tet*(A) variant conferring resistance to tigecycline was present in *E. hormaechei* G17. This strain harbored two resistance plasmids (pG17-1, 264,084 bp and pG17-2, 68,610 bp) and its E. coli transformant T_m_-G17_TGC_ one resistance plasmid (pTm-G17, 93,013 bp). The comparative analysis of pG17-1, pG17-2, and pTm-G17 showed that a *tet*(A) variant-carrying multiresistance gene cluster (~23 kb) originating from pG17-1 had integrated into pG17-2, forming the novel plasmid pTm-G17. In a first step, this multiresistance gene cluster was excised from pG17-1 by recombination of homologous sequences, including △Tn*As1* at both termini, thereby generating an unconventional circularizable structure (UCS). In a second step, this UCS integrated into pG17-2 via recombination between homologous sequences, including IS*26* present on both, the UCS and pG17-2, thereby giving rise to the new plasmid pTm-G17. In summary, a *tet*(A) variant conferring resistance to tigecycline was reported in *E. hormaechei*. Transfer of a *tet*(A) variant-carrying multiresistance gene cluster between plasmids occurred in a two-step recombination process, in which homologous sequences, including either △Tn*As1* or IS*26*, were involved.

**IMPORTANCE** Tigecycline is an important last-resort broad spectrum antimicrobial agent. This study describes the two-step recombination processes resulting in the transfer of the *tet*(A) variant gene between different plasmids in *E. hormaechei*, which depicts the role of recombination processes in the generation of UCSs and new plasmids, both carrying a *tet*(A) variant conferring resistance to tigecycline. Such processes enhance the dissemination of resistance genes, which is of particular relevance for resistance genes, such as the *tet*(A) variant. The presence and transmission of a *tet*(A) variant in *E. hormaechei* will compromise the efficacy of tigecycline treatment for *E. hormaechei* associated infection.

## INTRODUCTION

Enterobacter cloacae complex (ECC) is versatile, and the taxonomic status is constantly updated. There are more than 20 species and clades (without taxonomy terms) classified into it (1). It has been reported that Enterobacter hormaechei and *E. kobei* are the predominant ESBL-positive species accounting for community-acquired ECC strains collected from northern China (2). Nowadays the widespread existence of carbapenem-producing Enterobacteriaceae (CPE), has greatly limited the use of carbapenems in clinical treatment (3). However, the use of the alternative antimicrobial agents colistin and tigecycline is also restricted by newly emerged plasmid-mediated resistance genes, such as *mcr*, *tet*(A) and *tet*(X) variants (4–7).

Tigecycline belongs to the glycylcyclines, a new class of tetracycline derivatives. It is a broad-spectrum agent and exhibits bacteriostatic activity by binding to the 30S ribosomal subunit of bacteria (8). The mechanisms of resistance to tigecycline mainly include overexpression of efflux systems and enzyme modification. The AcrAB mutant efflux pump was the major mechanism of tigecycline resistance among Gram-negative bacteria, including Klebsiella pneumoniae and Enterobacter cloacae (9, 10). In recent years, it was reported that in Klebsiella pneumoniae, Enterobacter cloacae and Salmonella Typhimurium, the mutation of the *ramR* gene leaded to the increased expression of AcrAB efflux pump, which enhanced the resistance to various drugs, including tigecycline (11–13). In 2010, it was discovered in Salmonella that a variant of the efflux gene *tet*(A) can lead to low-level tigecycline resistance (14). The TetX enzyme, which modifies tigecycline, was first reported in 2005 (15). Two plasmid-mediated high-level tigecycline resistance genes *tet*(X3) and *tet*(X4) were identified in 2019 (6, 7). The latest *tet*(X) variants reported in 2021 include 27 new variants, from *tet*(X18) to *tet*(X44), which were identified in Riemerella anatipestifer (16), and *tet*(X15) in Acinetobacter variabilis (17).

Insertion sequences (ISs) seem to play an important role in the dissemination of antimicrobial resistance genes in both Gram-positive and Gram-negative bacteria. If two identical ISs, located in the same orientation, bracket DNA sequences, these two ISs can recombine and form translocatable units (TUs), which contain one copy of the IS and the region between the two ISs, which often comprises resistance genes (18, 19). These TUs may then integrate into plasmids or chromosomal sites, thereby fostering the dissemination of resistance genes (19). More rarely, it has been reported that circular structures can also be formed through recombination of homologous sequences at both termini that lack recombinase genes (20–25). These structures were tentatively referred to as unconventional circularizable structures (UCSs) in 2013 (26). The existence of these structures broadens the way for the spread of resistance genes.

In this study, the contribution of a *tet*(A) variant to tigecycline resistance in *E. hormaechei* was investigated. In addition, the transmission of this variant was analyzed with regard to the formation of an UCS and the integration of this UCS into a new plasmid background.

## RESULTS AND DISCUSSION

### Two multidrug resistance plasmids harboring UCS in *E. hormaechei* G17.

**FIG 1:**
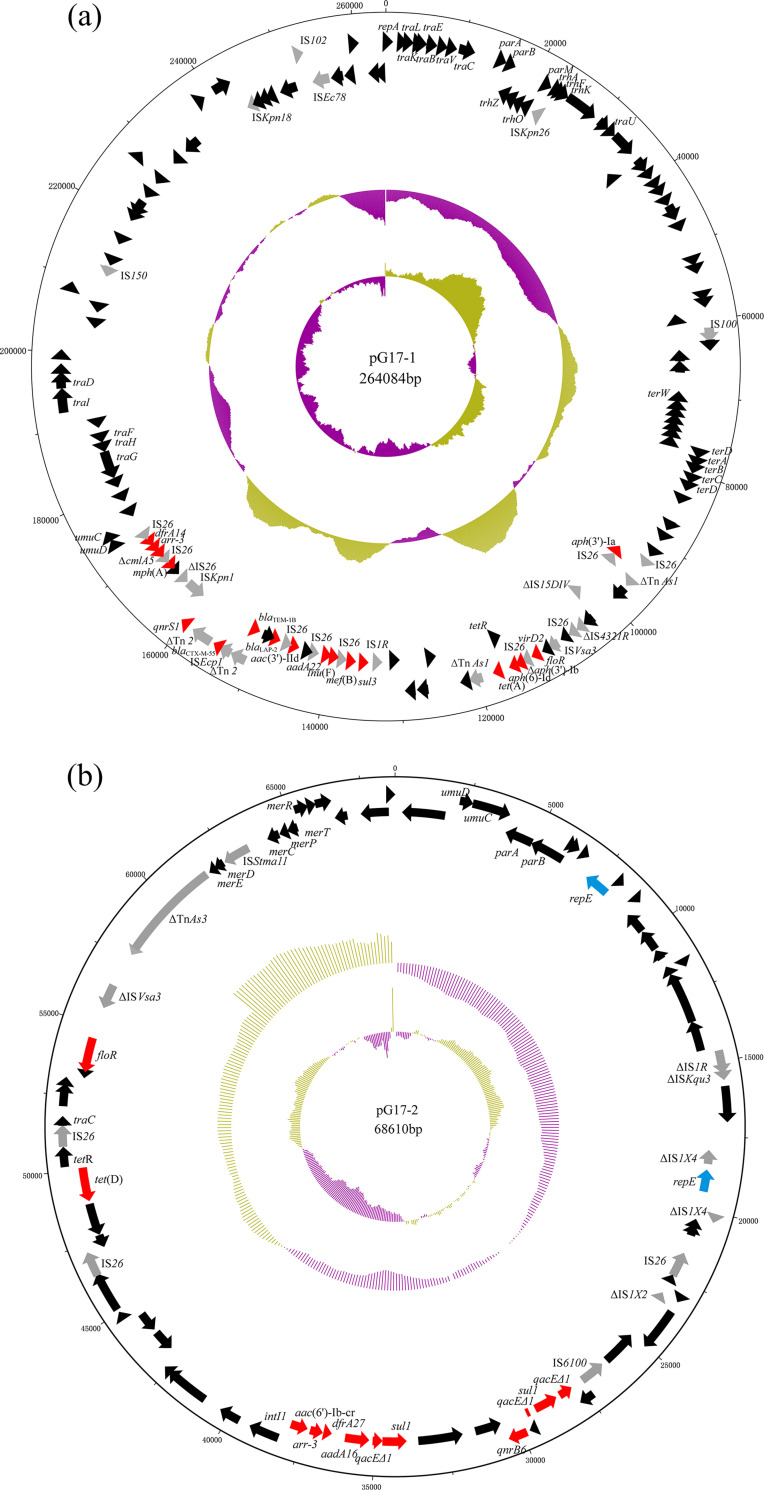
The structures of plasmids pG17-1 (a) and pG17-2 (b) *from E. hormaechei* G17. The circles in (a) and (b) depict (from the outside to inside): (i) the size scale in bp; (ii) the positions of predicted coding sequences transcribed in the clockwise orientation; (iii) the positions of predicted coding sequences transcribed in the counterclockwise orientation; (iv) the GC content plotted against 50%, with blue indicating >50% and green indicating <50%; and (v) GC skew [(G- C)/(G+C)] in a 10,000 bp window. Genes are color-coded, depending on functional annotations: red, antimicrobial resistance; gray, transposition; black, other genes and plasmid replication.

**FIG 2:**
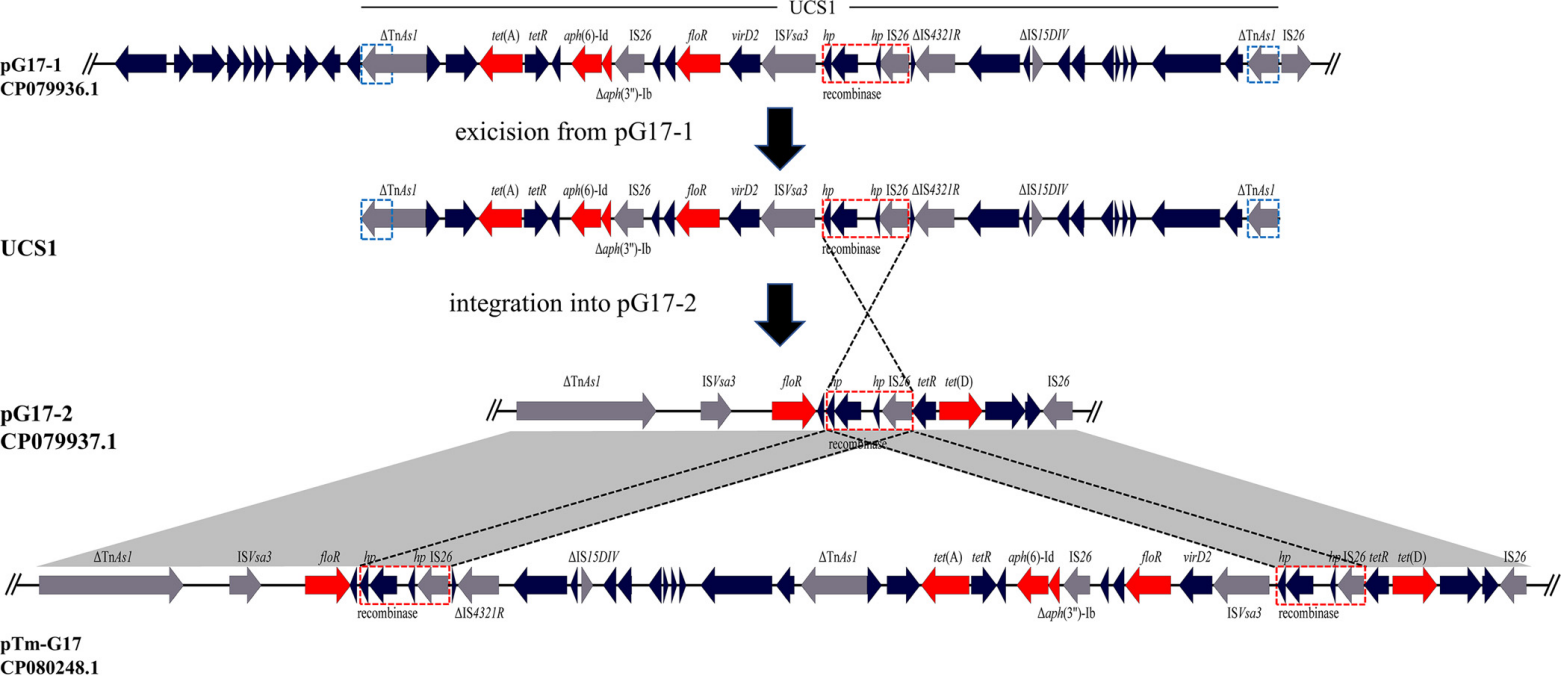
Transfer of a *tet*(A) variant-carrying multiresistance gene cluster between the plasmids via a two-step recombination process revealed by the comparative analysis of the relevant regions of plasmids pG17-1, pG17-2, and pTm-G17. UCS1 was formed by the recombination between a pair of similar repeated sequences (blue dashed boxes), covered by a horizontal straight line. UCS1 integrated into pG17-2 via another pair of homologous sequences (red dashed boxes). The integration process was shown in the dotted line. The genes are shown as arrows, with the arrowhead indicating the direction of transcription. Antimicrobial resistance genes are shown in red, the transposition-related genes in gray and other genes in black. The gray-shaded area indicates regions of >99% nucleotide sequence identity.

*E. hormaechei* strain G17 contained two plasmids, designated pG17-1 and pG17-2, which were 264,084 bp and 68,610 bp in size, respectively (Fig. 1). Both of them were hybrid plasmids, with pG17-1 belonging to the incompatibility group IncHI2/IncHI2A, and pG17-2 to IncFIA(HI1)/IncR. pG17-1 harbored a tigecycline resistance-mediating *tet*(A) gene variant, the rifamycin resistance gene *arr-3*, the phenicol resistance gene *floR*, the aminoglycoside resistance genes *aph*(6)-Id, *aac*(3)-IId, *aac*(3′)-Ia, and *aadA22*, the sulfonamide resistance gene *sul3*, the trimethoprim resistance gene *dfrA14*, the lincomycin resistance gene *lnu*(F), the quinolone resistance gene *qnrS1*, the β-lactam resistance genes *bla*_CTX-M-55_, *bla*_LAP-2_, and *bla*_TEM-1B_, as well as the macrolide resistance genes *mph*(A) and *mef*(B) (Fig. 1a). As shown in Fig. 2, the *tet*(A) variant was flanked by copies of homologous sequences, including △Tn*AS1*, which can recombine and form an UCS, designated UCS1. This was also evidenced by PCR using the primers listed in Table S1 and Sanger sequencing (Fig. S1).

pG17-2 carried the aforementioned resistance genes *arr-3* and *floR*, but also the tetracycline resistance gene *tet*(D), the aminoglycoside resistance genes *aadA16* and *aac*(6’)*-Ib-cr*, the sulfonamide resistance gene *sul1*, the trimethoprim resistance gene *dfrA27*, and the quinolone resistance gene *qnrB6* (Fig. 1b). The quinolone resistance gene *qnrB6* located downstream of the class I integron was flanked by two copies of homologous sequences, including *qacEΔ1* and *sul1* genes on pG17-2. Each of these copies was 1,181 bp in size and the upstream and downstream copies were identical in their nucleotide sequences. PCR revealed that UCS2 consisting of the *qnrB6* gene and one copy of hybrid sequence derived from recombination between the homologous sequences, including *qacEΔ1* and *sul1* genes was formed (Fig. S1), suggesting that the UCS2 containing *qnrB6* is active.

### *tet*(A) variant on pG17-1 in *E. hormaechei* was identified to confer resistance to tigecycline.

In order to verify whether the *tet*(A) variant in *E. hormaechei* G17 confers resistance to tigecycline, *E. hormaechei* G17 was used as the donor in conjugation and transformation experiments. No transconjugant was obtained in multiple attempts when using 0.25 mg/L tigecycline for screening. However, transformants were obtained on screening media supplemented with 0.25 mg/L tigecycline or 4 mg/L tetracycline, respectively. Two different transformants, designated E. coli T_m_-G17_TGC_ (selected from media supplemented with 0.25 mg/L tigecycline) and E. coli T_m_-G17_TET_ (selected from media supplemented with 4 mg/L tetracycline), respectively, were chosen for further analysis. The transformant E. coli T_m_-G17_TGC_ displayed resistance to both tigecycline (8-fold MIC increase) and tetracycline (>128-fold MIC increase) compared with the recipient E. coli DH5α. However, the transformant E. coli T_m_-G17_TET_ displayed no increase in the MIC of tigecycline, but a >64-fold increase in the MIC of tetracycline, compared with the recipient E. coli DH5α (Table 1).

**TABLE 1:** MICs of *E. hormaechei* G17, transformant T_m_-G17_TET_, transformant T_m_-G17_TGC_ and E. coli DH5α

Strains	MICs (mg/L)^*a*^						
	TET	TGC	CHL	FEP	TMP	CAZ	RIF
*E. hormaechei* G17	256	8	>512	128	2	512	>512
T_m_-G17_TGC_	128	1	256	<1	2	<1	>512
T_m_-G17_TET_	64	0.125	128	<1	2	<1	512
E. coli DH5α	<1	0.125	2	<1	<1	<1	8

a TET, tetracycline; TGC, tigecycline; CHL, chloramphenicol; FEP, cefepime; TMP, trimethoprim; CAZ, ceftazidime; RIF, rifampin.

PCR analysis revealed that the transformant E. coli T_m_-G17_TGC_ was not only positive for the conserved backbone sequence on pG17-2, but also positive for both, the *tet*(A) variant and *tet*(D), whereas the transformant E. coli T_m_-G17_TET_ was only positive for the conserved backbone sequence on pG17-2 and *tet*(D). S1-PFGE revealed that *E. hormaechei* G17 contained two plasmids (pG17-1, ~264 kb and pG17-2, ~68 kb), E. coli T_m_-G17_TGC_ and E. coli T_m_-G17_TET_ contained an ~93 kb plasmid (pTm-G17) and an ~68 kb plasmid (pG17-2), respectively (Fig. S2). Southern blot hybridization showed that the *tet*(A) variant was originally located on the ~264 kb pG17-1 in *E. hormaechei* G17, but appeared on the ~93 kb plasmid pTm-G17 in E. coli T_m_-G17_TGC_ after transformation, suggesting the *tet*(A) variant can be transferred between the plasmids. Protein sequence analysis with the software DNAMAN8.0 indicated that the Tet(A) in *E. hormaechei* G17 in this study had the same amino acid sequence as the previously reported Tet(A) variant in Klebsiella pneumoniae KP267 (Fig. S3), which had been proved to confer resistance to tigecycline (4). There are seven amino acid substitutions occurring in the deduced amino acid sequence of the Tet(A) from *E. hormaechei* G17 (Identity, 99.42%), compared with the reference RP1 Tet(A) from E. coli plasmid RP1 (27, 28). Of them, the key amino acid substitutions 201-SFV-203 in Tet(A) from RP1 to 201-ASF-203 in Tet(A) from pGF17 and K. pneumoniae KP267 had been considered to be involved in tigecycline resistance (28).

### The *tet*(A) variant was transferred between the plasmids via a two-step recombination process.

The comparative analysis of plasmids pG17-1, pG17-2, and pTm-G17 showed that a *tet*(A) variant-carrying multiresistance gene cluster (~23 kb), originating from pG17-1 had integrated into pG17-2, forming the novel plasmid pTm-G17 (~93 kb, Fig. 2). The specific integration process is as follows: in a first step, the *tet*(A) variant-carrying multiresistance gene cluster was looped out from plasmid pG17-1 by recombination between homologous sequences of 849 bp, which contained a truncated copy of transposon Tn*As1*, designated △Tn*As1.*These homologous sequences were present at both termini of the cluster (Fig. 2), and recombination between them formed UCS1. In a second step, the *tet*(A) variant-carrying UCS1 integrated into pG17-2 via another pair of homologous sequences which were present on both the UCS1 and plasmid pG17-2. These homologous sequences had a size of 2,470 bp and contained a copy of IS*26* (Fig. 2). In pTm-G17, the pG17-1-derived multiresistance gene cluster was bracketed by copies of the homologous sequences containing IS*26*. Where and when plasmid pTm-G17 was generated is unknown. However, since both partner plasmids, pG17-1 and pG17-2, are required for the formation of pTm-G17, it is likely that pTm-G17 was already formed in the donor strain and was present there at a very low copy number, too low to be detected by PFGE and subsequent hybridization. When transformed into E. coli, this plasmid became the only plasmid in the new host and was now detectable at the regular copy number. The two-step recombination processes detailed above resulted in the transfer of the *tet*(A) variant gene between different plasmids. Such processes enhance the dissemination of resistance genes, which is of particular relevance for resistance genes, such as the *tet*(A) variant gene which mediates resistance not only to tetracycline but also to tigecycline.

## MATERIALS AND METHODS

### Bacterial strains and antimicrobial susceptibility testing.

*E. hormaechei* strain G17 was obtained from the liver of a diseased duck in a traditional duck farm in Henan province, China, in 2019. It was identified and stored in our lab. Antimicrobial susceptibility testing (AST) was conducted by broth microdilution according to the recommendations of the Clinical and Laboratory Standards Institute (CLSI) for tetracycline, tigecycline, chloramphenicol, cefepime, trimethoprim, ceftazidime and rifampin (29). The tigecycline MICs were interpreted according to the recommendations of EUCAST (https://www.eucast.org/ast_of_bacteria/). E. coli ATCC 25922 served as the quality control strain in AST.

### Transfer experiments.

Conjugation and transformation experiments were performed using *E. hormaechei* G17 as the donor, and E. coli DH5α as the recipient. For the screening of the transconjugants, LB agar was supplemented with 0.25 mg/L tigecycline. For the screening of the transformants, LB agar was supplemented with 0.25 mg/L tigecycline or 4 mg/L tetracycline, respectively. Colonies that grew on these selective plates at 37°C after incubation for 24 h were further confirmed by AST as well as PCR amplification followed by Sanger sequencing and analysis of the *tet*(A) gene.

### PCR analysis.

The resistance gene *tet*(A) variant, the conserved backbone sequence on the plasmids pG17-1 or pG17-2, and the presence of UCSs were detected by PCR using the primers and conditions listed in Table S1 (available as supplemental material). All the PCR products were subjected to Sanger sequencing.

### S1-PFGE and Southern blot hybridization.

The genomic DNA of the donor *E. hormaechei* G17, the recipient E. coli DH5α and two transformants E. coli T_m_-G17_TGC_ and E. coli T_m_-G17_TET_ in agarose gel plugs were digested with S1 endonuclease (TaKaRa, Dalian, China), separated by PFGE as previously described (30), transferred to Amersham Hybond-N+ membranes (GE Healthcare), and hybridized with a digoxigenin-labeled *tet*(A) variant probe *(tet[A] [nt 7292–7676; X61367.1])*. Detection was performed by using a DIG-DNA labeling and detection kit (Roche Diagnostics, Germany).

### Sequencing and sequence analysis.

The whole-genome DNA of *E. hormaechei* strain G17 and the transformant E. coli T_m_-G17_TGC_ were sequenced by using the PacBio RS and Illumina MiSeq platforms (Shanghai Personal Biotechnology Co., Ltd., China). The PacBio sequence reads were assembled with HGAP4 and CANU (Version 1.6), corrected by Illumina MiSeq with pilon (Version 1.22). The prediction of ORFs and their annotations were performed using Glimmer 3.0. The blast software was used following the procedures at https://blast.ncbi.nlm.nih.gov. The Illumina short read sequences and PacBio long read sequences were mapped to the spliced sequences by BWA (Version: 0.7.17-r1188), and the chromosome and plasmid depths were calculated by mosdepth 0.3.3. In G17 strain, the read depths of chromosome and three plasmids by short sequence alignment were 2.71, 27.47, 16.01, and 140, respectively, and the read depths of long sequence alignment were 31.38, 13.94, 13.99, and 30.20, respectively. The read depths of chromosome and a plasmid in T_m_-G17_TGC_ by short sequence alignment were 258.85 and 470.75, respectively, and the read depths of long sequence alignment were 325.56 and 264.78, respectively.

### Data availability.

The sequences of three plasmids pG17-1, pG17-2 and pTm-G17 and two chromosomes from G17 and T_m_-G17_TGC_ determined in this study have been deposited in GenBank under accession numbers CP079936.1, CP079937.1, CP080248.1, CP079939.1, and CP080247.1, respectively.
