# Supplementary material for: Studies on the Transmission of a Tigecycline Resistance-Mediating tet(A) Gene Variant from Enterobacter hormaechei via a Two-Step Recombination Process
Source: Microbiol Spectr. 2022 May 17;10(3):e00496-22. doi: 10.1128/spectrum.00496-22 (PMC9241890; doi:10.1128/spectrum.00496-22)
Supplement: SUPPLEMENTAL FILE 1 — Supplemental material. Download spectrum.00496-22-s001.pdf, PDF file, 0.4 MB [file spectrum.00496-22-s001.pdf]

The Supplementary data

TABLE S1 PCR primers and conditions used in this study

Name	Primer designation	Sequence (5 -3)	Description	Amplicon size (bp)
UCS1	UCS1-fw	AGCGCCGTGCCATAAAAGA	Used to detect the UCS formed by two copies of ΔTn*As1* gene.	2137
	UCS1-rv	ATTATCAGCCACATCTACCCAC		
UCS2	UCS2-fw	TCACGCCAGTTAGTATCGAG	Used to detect the UCS formed by two copies of *qacEΔ1* and *sul1* gene.	3615
	UCS2-rv	CGCCAATATGACATCTGCAA		
tet(A) variant	*tet*(A)-fw	CTCAACCCGCTCGCTTCGTTC	Used to detect the resistance gene *tet*(A) variant.	395
	tet(A)-rv	ACCTGCCTGGACAACATTGCT		
pG17-1-b	pG17-1-b-fw	CAACGCAACACCAGACCAG	Used to detect the conserved sequence on the pG17-1 plasmid backbone.	1715
	pG17-1-b-rv	GCTTACTTTCCGGACGACT		
pG17-2-b	pG17-2-b-fw	AAATGGCAGGGACAGGTGA	Used to detect the conserved sequence on the pG17-2 plasmid backbone.	2380
	pG17-2-b-rv	CGATAGGCCCGATCTTGGT		

(a)

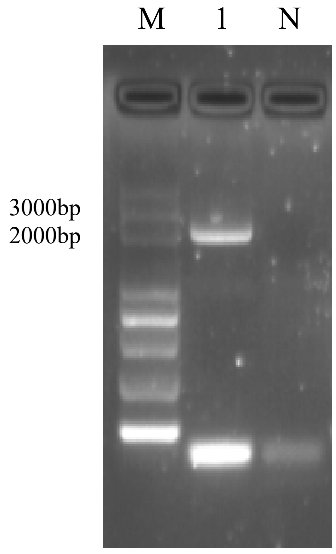

(b)

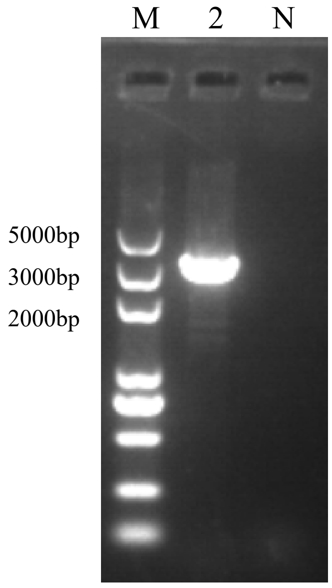

FIG S1 The PCR amplification for the UCS1 (a) and UCS2 (b) in *E. hormaechei* G17**.** The PCR amplification for the UCSs.

M. DL5000marker; Lanes 1, 2.The PCR amplification for the UCS1 and UCS2; N. Negative control.

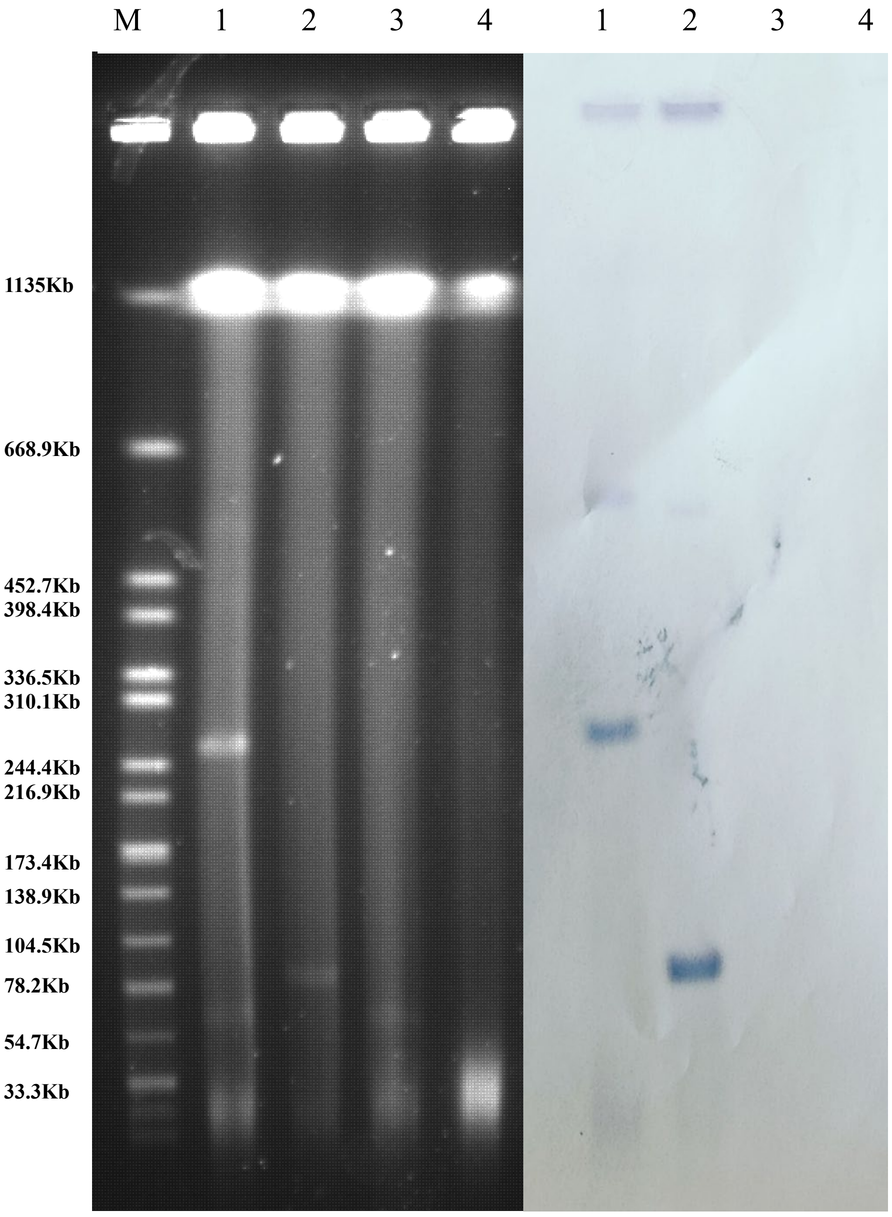

FIG S2 Detection of *tet*(A)_variant-carrying plasmids in *E. hormaechei* G17 and its transformants *E. coli* by S1-PFGE (a) and Southern blot hybridization with a *tet*(A) variant probe (b). Lane 1, *E. hormaechei* G17; Lane 2, *E. coli* Tm-G17$_{TGC}$; Lane 3, *E. coli* Tm-G17$_{TET}$; Lane 4, *E. coli* DH5α; M, H9812.

```
ORIGIN
G17_tet(A)_v.txt    MKPNRPLIVILSTVALDAVGIGLIMPVLPGLLRDLVHSND     40
KP267_tet(A)_v.txt  ----------------------------------------     40
RP1_tet(A)_ref.txt  ----i-----------------------------------     40

G17_tet(A)_v.txt    VTAHYGILLALYALMQFACAPVLGALSDRFGRRPVLLVSL     80
KP267_tet(A)_v.txt  ----------------------------------------     80
RP1_tet(A)_ref.txt  --------------v-------------------i-----     80

G17_tet(A)_v.txt    AGAAVDYAIMATAPFLWVLYIGRIVAGITGATGAVAGAYI    120
KP267_tet(A)_v.txt  ----------------------------------------    120
RP1_tet(A)_ref.txt  ---t------------------------------------    120

G17_tet(A)_v.txt    ADITDGDERARHFGFMSACFGFGMVAGPVLGGLMGGFSPH    160
KP267_tet(A)_v.txt  ----------------------------------------    160
RP1_tet(A)_ref.txt  ----------------------------------------    160

G17_tet(A)_v.txt    APFFAAAALNGLNFLTGCFLLPESHKGERRPLRREALNPL    200
KP267_tet(A)_v.txt  ----------------------------------------    200
RP1_tet(A)_ref.txt  ----------------------------------------    200

G17_tet(A)_v.txt    ASFRWARGMTVVAALMAVFFIMQLVGQVPAALWVIFGEDR    240
KP267_tet(A)_v.txt  ----------------------------------------    240
RP1_tet(A)_ref.txt  sfv-------------------------------------    240

G17_tet(A)_v.txt    FHWDATTIGISLAAFGILHSLAQAMITGPVAARLGERRAL    280
KP267_tet(A)_v.txt  ----------------------------------------    280
RP1_tet(A)_ref.txt  ----------------------------------------    280

G17_tet(A)_v.txt    MLGMIADGTGYILLAFATRGWMAFPIMVLLASGGIGMPAL    320
KP267_tet(A)_v.txt  ----------------------------------------    320
RP1_tet(A)_ref.txt  ----------------------------------------    320

G17_tet(A)_v.txt    QAMLSRQVDEERQGQLQGSLAALTSLTSIVGPLLFTAIYA    360
KP267_tet(A)_v.txt  ----------------------------------------    360
RP1_tet(A)_ref.txt  ----------------------------------------    360

G17_tet(A)_v.txt    ASITTWNGWAWIAGAALYLLCLPALRRGLWSGAGQRADR     399
KP267_tet(A)_v.txt  ---------------------------------------     399
RP1_tet(A)_ref.txt  ---------------------------------------     399
```

FIG S3 Amino acid substitutions occurring in the deduced amino acid sequences of the Tet(A) protein from tigecycline-resistant *E. hormaechei* G17, compared with the reported Tet(A) variant of plasmid pKPC-CR-HvKP267 from tigecycline-resistant *K. pneumoniae* KP267 (accession number MG053313) and the reference Tet(A) of plasmid RP1 from tigecycline-susceptible *E. coli* (accession number X00006.1).
